# Interactive Effects of Glucocorticoids and Cytochrome P450 Polymorphisms on the Plasma Trough Concentrations of Voriconazole

**DOI:** 10.3389/fphar.2021.666296

**Published:** 2021-05-25

**Authors:** Su-jie Jia, Ke-qin Gao, Pan-hao Huang, Ren Guo, Xiao-cong Zuo, Qing Xia, Shuang-yao Hu, Zhen Yu, Yue-liang Xie

**Affiliations:** ^1^ Department of Pharmacy, The Third Xiangya Hospital of Central South University, Changsha, China; ^2^ Department of Pharmacy and Center of Clinical Pharmacology, The Third Xiangya Hospital, Central South University, Changsha, China; ^3^ Department of Clinical Pharmacy, Weifang People’s Hospital, Weifang, China; ^4^ Zunyi Medical College, Zunyi, China; ^5^ Department of Pharmacy, Kangya Hospital, Yiyang, China

**Keywords:** glucocorticoids, CYP2C19, CYP3A4, CYP3A5, voriconazole

## Abstract

**Aims:** To explore the interactive influence of glucocorticoids and cytochrome P450 (CYP450) polymorphisms on voriconazole (VRC) plasma trough concentrations (C_min_) and provide a reliable basis for reasonable application of VRC.

**Methods:** A total of 918 VRC C_min_ from 231 patients was collected and quantified using high-performance liquid chromatography in this study. The genotypes of *CYP2C19*, *CYP3A4*, and *CYP3A5* were detected by DNA sequencing assay. The effects of different genotypes and the coadministration of glucocorticoids on VRC C_min_ were investigated. Furthermore, the interactive effects of glucocorticoids with CYP450s on VRC C_min_ were also analyzed.

**Results:** The median C_min_ of oral administration was lower than that of intravenous administration (1.51 vs. 4.0 mg l^−1^). Coadministration of glucocorticoids (including dexamethasone, prednisone, prednisolone, and methylprednisolone) reduced the VRC C_min_/dose, respectively, among which dexamethasone make the median of the VRC C_min_/dose ratio lower. As a result, when VRC was coadministrated with glucocorticoids, the proportion of VRC C_min_/dose in the subtherapeutic window was increased. Different CYP450 genotypes have different effects on the C_min_/dose of VRC. Mutations of CYP2C19*2 and *3 increased C_min_/dose of VRC, while CYP2C19*17 and *CYP3A4* rs4646437 polymorphisms decreased C_min_/dose of VRC. The mutation of *CYP3A5* has no significant effect. Furthermore, CYP2C19*17 mutants could strengthen the effects of glucocorticoids and decrease VRC C_min_/dose to a larger extent.

**Conclusion:** Our study revealed that glucocorticoids reduced the C_min_/dose levels of VRC and different SNPs of CYP450 have different effects on the C_min_/dose ratio of VRC. Glucocorticoids and CYP2C19*17 mutants had a synergistic effect on reducing VRC C_min_/dose. The present results suggested that when VRC is combined with glucocorticoids, we should pay more attention to the clinical efficacy of VRC, especially when CYP2C19*17 mutants exist.

## Introduction

With the increasing incidence of malignant tumors, acquired immune deficiency syndrome, and organ transplantation, the morbidity and mortality of invasive fungal infections are rising and have gradually become one of the major threats to human health in recent years (Sanguinetti et al., 2019). Voriconazole (VRC) is a second-generation triazole antifungal drug, inhibiting the activity of cytochrome P450 (CYP450)-dependent 14α-sterol demethylase so that lanosterol cannot be converted into its 14α-demethylated products in order to prevent ergosterol biosynthesis and change the integrity of membrane, which leads to the rupture and death of fungal cells (Naithani and Kumar, 2005). VRC has a spacious antibacterial spectrum, including *Candida*, *Cryptococcus neoformans*, *Aspergillus*, *Fusarium*, *Histoplasma*, and the other fungi (Thompson and Lewis, 2010). So far, VRC is widely used to prevent and treat invasive fungal diseases and recommended by guidelines as the first-line therapy (Ullmann et al., 2018).

In clinical application, it was gradually found that VRC had great individual differences. The steady-state trough concentrations of VRC varied greatly, which may be related with its nonlinear pharmacokinetics and many other clinical factors (Karlsson et al., 2009). Studies have shown that supratherapeutic VRC C_min_ was associated with its hepatotoxicity, nervous system disorders, and visual disturbance, while subtherapeutic VRC C_min_ may lead to treatment failures (Jin et al., 2016). It has been proven that VRC is of narrow therapeutic window (the recommended range is between 0.5–1.5 and 5–5.5 mg l^−1^) and therapeutic drug monitoring (TDM) (Ashbee et al., 2014) is an effective way to VRC-individualized medication (Chen et al., 2018a; Ullmann et al., 2018). As a result, it is very important to clarify the factors affecting VRC concentrations in clinical practice. It is found that intra- and inter-individual variabilities of VRC C_min_ depend on age, actual body weight, CYP450 polymorphisms including CYP2C19, CYP3A4, and CYP3A5, liver functions, hypoproteinemia, inflammation, and drug–drug interactions (DDIs) (Chawla et al., 2015; Gautier-Veyret et al., 2015). Among them, CYP450 polymorphisms and CYP-mediated drug interactions are important determinants of intra- and inter-individual variabilities of VRC.

Glucocorticoids such as dexamethasone, prednisone, prednisolone, and methylprednisolone are widely coadministered with VRC in patients with hematological malignancies or solid organ transplantations. Naturally, there were some studies on the effects of concomitant medication of glucocorticoids and VRC. However, the results of different researches are inconsistent. It is a hot spot of controversy whether concomitant with glucocorticoids affects VRC C_min_ and whether different glucocorticoids (ie., dexamethasone, prednisone, prednisolone, and methylprednisolone) have same effects on VRC concentrations (Eiden et al., 2010; Dolton et al., 2012; Gautier-Veyret et al., 2015; Cojutti et al., 2016; Li et al., 2017; Blanco-Dorado et al., 2020), and the mechanism of this interaction is still unclear. In general, glucocorticoids are strong inducers of CYP2C9, CYP2C11, CYP2C19, CYP3A4, CYP3A5, and CYP3A7 (Iber et al., 1997; Chen et al., 2003; Zhou et al., 2009; Dvorak and Pavek, 2010; Matsunaga et al., 2012; Matoulkova et al., 2014), which leads to a C_min_ decrease of drugs that are metabolized primarily by these CYP450s. VRC is mainly metabolized by CYP450s, thus may have DDIs with glucocorticoids. Due to the inconsistent results of previous studies, the purpose of this experiment is mainly focused on the effects of glucocorticoids on VRC C_min_.

VRC is metabolized mainly by CYP450 enzymes and the effects of CYP450 polymorphisms on VRC C_min_ have been widely discussed. Among them, CYP2C19, CYP3A4, and CYP3A5 are considered to be highly correlated with VRC metabolism (Iber et al., 1997; Chen et al., 2003; Zhou et al., 2009; Dvorak and Pavek, 2010; Matsunaga et al., 2012; Matoulkova et al., 2014). VRC is metabolized predominantly by CYP2C19, and variant *CYP2C19* alleles contribute to wide inter-patient variabilities of VRC serum concentrations (Moriyama et al., 2017). Recently, *CYP3A4* and *CYP3A5* polymorphisms were demonstrated to affect VRC C_min_ by some studies, while other studies identified that polymorphisms of *CYP3A4* and *CYP3A5* have no significant influences on VRC C_min_. Hence, the effects of *CYP3A4* and *CYP3A5* polymorphisms on VRC need to be further studied (Gautier-Veyret et al., 2015; Gautier-Veyret et al., 2016). In *CYP2C19* mutational subjects, the pharmacokinetics of VRC did not change compared to *CYP2C19* wild type ones, so the influence of *CYP2C9* polymorphisms on VRC was not obvious (Geist et al., 2006). Therefore, only the influences of *CYP2C19*, *CYP3A4*, and *CYP3A5* polymorphisms on VRC concentrations were emphasized in our study. These CYP450 enzymes confirmed to affect VRC metabolism that can be induced by glucocorticoids, which indicate the potential DDIs between VRC and glucocorticoids.

Therefore, the objectives of this study are to identify the influences of four glucocorticoids (dexamethasone, prednisone, prednisolone, and methylprednisolone) on VRC C_min_, and to further explore the effects of CYP450 polymorphisms on the interaction between glucocorticoids and VRC.

## Materials and Methods

### Patients and Data Collection

This retrospective study was performed at the Third Xiangya Hospital of Central South University, Changsha, China. Patients underwent TDM of VRC concentrations were recruited from January 2016 to June 2018. The inclusion criteria were that patients aged 18 years or older underwent TDM of VRC plasma concentrations at the trough level under steady state (Gautier-Veyret et al., 2015). Patients received concomitant drugs that were CYP inducers such as phenobarbital, rifampin, phenytoin, and carbamazepine or CYP inhibitors such as cimetidine and erythromycin were excluded (Yan et al., 2018). For each patient, the following data were collected: demographics (age, gender, and actual body weight), clinical data (underlying disease) and VRC therapy records (daily dosage, dosage adjustment, C_min_, and route of administration), and concomitant medications.

The design of this research was completely conformed to the principles of the Helsinki Accords, and this study was approved by the Ethics Research Committee of the Third Xiangya Hospital of Central South University (No: 2017-S220). All subjects signed the informed consent that DNA was extracted from residual blood samples from VRC concentration analyses for laboratory testing.

### Determination of Plasma VRC Concentration

The blood samples were collected 0–30 min before administration until at least 3 days of the scheduled treatment, and all the unsteady state concentrations of VRC were removed. VRC plasma concentrations were measured by a validated high-performance liquid chromatography method (Yan et al., 2018). Briefly, samples were injected into a 2-dimensional chromatographic system. In the first step, samples were pre-separated by a perfusion chromatography column before being eluted and transferred to an analytical column. Finally, compounds were detected by a multi-channel rapid-scanning UV–VIS detector. Precision and accuracy were assessed by performing replicate analyses of quality control samples against calibration standards. Intra- and inter-assay coefficients of variation were always <5%. The plasma drug standard curve ranged from 0.1 to 20 mg l^−1^.

### Genotyping Assay

Genotyping was performed retrospectively on residual blood samples from VRC concentration analyses. DNA was extracted from peripheral leukocytes by the TIANamp Genomic DNA Kit (TianGen Biotech, Beijing, China). The quality and quantity of DNA were checked with the NanoDrop 2000 spectrophotometer (Thermo Scientific, Illkirch, France). The DNA samples were stored in −80°C until genotype detection. Genotyping adopted by the Sanger DNA sequencing method with an ABI3730xl-full automatic sequencing instrument (ABI Co.) from Boshang Biotechnology Co. Ltd. in Shanghai. *CYP2C19* genotyping was performed for the *2, *3, and *17 alleles. Three single-nucleotide polymorphisms (SNPs) (rs35599367 and rs4646437 in *CYP3A4*, and rs776746 in *CYP3A5*) that were known commonly to affect the plasma VRC concentrations were also genotyped in the present study.

### Statistical Analysis

The statistical analyses were performed with SPSS 22.0 software (IBM SPSS, Inc., Chicago, IL, United States). The quantitative data were expressed as the mean ± standard deviation (SD), while the counting data in frequency and percentage. The Kolmogorov–Smirnov test was used for normality of measurement data. Non-normal distributed data were represented by median and interquartile difference (IQR). Nonparametric tests were used to compare non-normal distribution data (Mann–Whitney test for two groups, Kruskal–Wallis test for at least three groups, and Wilcoxon rank sum test for comparisons of paired designs). The chi-squared test was used to compare counting data. Interaction between CYP450 genotypes and glucocorticoids was analyzed by the Scheirer–Ray–Hare test. *p* < 0.05 was considered statistically significant.

## Results

### Patient Characteristics

A total of 231 patients were enrolled in this study. Of the 231 patients, 134 (58.0%) were male and 97 (42.0%) were female. The mean age and weight of patients were 51.47 ± 17.55 years and 57.24 ± 10.98 kg, respectively. The top three underlying diseases in VRC-treated patients were hematological malignancy (*n* = 137, 59.3%), pulmonary diseases (*n* = 33, 14.3%), and septic shock (*n* = 18, 7.8%). The most common hematological malignancies were leukemia (*n* = 93, 40.3%). Among 231 patients, 159 patients had genetic tests and 103 patients had the concomitant administration of glucocorticoids. The patient demographics and characteristics in this study are summarized in Table 1.

**TABLE 1:** Characteristics of 231 patients included in the study.

Characteristics	Patient, N (%)
Total number of patients	231
Male, *n* (%)	134 (58.0%)
Age (year)	51.47 ± 17.55
Weight (kg)	57.24 ± 10.98
BMI	21.39 ± 2.78
Underlying disease, n (%)	
Hematological malignancy	**137 (59.3%)**
Leukemia	93 (40.3%)
Multiple myeloma	17 (7.4%)
Lymphoma	12 (5.2%)
Aplastic anemia	7 (3.0%)
Other	8 (3.5%)
Solid organ transplantation	**14 (6.1%)**
Solid malignancy	**7 (3.0%)**
Pulmonary disease	**33 (14.3%)**
COPD	16 (6.9%)
Bronchitis	11 (4.8%)
Other	6 (5.2%)
Septic shock	**18 (7.8%)**
Liver disease	**7 (3.0%)**
Other	**15 (6.5%)**
Number of genetic tests patient	**159 (68.83%)**
Number of patient concomitant with glucocorticoids	**103 (44.59%)**

### VRC Trough Concentration Therapeutic Drug Monitoring

A total of 918 VRC plasma steady-state trough concentrations from 231 patients were included in this study. The daily dose of VRC ranges from 100 to 800 mg. VRC C_min_ was adjusted on daily dose (for C_min_/dose ratio and C/D ratio) for overcoming the effect of dose (Gautier-Veyret et al., 2017; Shao et al., 2017). For example, the VRC daily dose for a patient is 400 mg d^−1^ and the C_min_ is 1,600 mg l^−1^. Thus, the C_min_/dose ratio of this patient is expressed as 4 mg l^−1^/mg·d^−1^. As shown in Table 2, grading criteria of VRC C_min_ were based on the individualized medication of VRC guidelines issued by the Chinese Pharmacological Society (Chen et al., 2018a). Similar to previous reports (Zeng et al., 2020), VRC C_min_ were mostly the concentration of oral administration (86.6%). Meanwhile, compared with oral administration, VRC C_min_ and the C_min_/dose ratio were generally higher in intravenous administration (*p* < 0.001). 76.5% C_min_/dose were under the therapeutic window of VRC ([1.25, 12.5] mg·l^−1^/mg d^−1^). Higher proportion of supratherapeutic VRC C_min_/dose (>12.5 mg l^−1^/mg d^−1^) and lower proportion of subtherapeutic VRC C_min_/dose (<1.25 mg l^−1^/mg d^−1^) were found in intravenous administration compared to oral administration (33.3% *vs.* 8.4% and 4.9% *vs.* 12.8%). These results revealed that we should pay more attention to the safety of supratherapeutic VRC C_min_ in intravenous administration, while treatment failure results from subtherapeutic VRC C_min_ in oral administration, respectively.

**TABLE 2:** VRC plasma trough concentration included in the study.

Parameter	All (*n* = 918)	Oral (*n* = 795, 86.6%)	Intravenous (*n* = 123, 13.4%)	*p*
C_min_ (mg l^−1^)				**<0.001**
Median (IQR)	1.64 (0.90, 3.00)	1.51 (0.85, 2.60)	4.00 (2.30, 5.80)	
Range	0.04–20.4	0.04–20.4	0.08–16.17	
C_min_ level, *n* (%)^a^				**<0.000**
>0.5	105 (11.4%)	99 (12.5%)	6 (4.9%)	
[0.5, 5]	714 (77.8%)	639 (80.4%)	75 (61.0%)	
>5	99 (10.8%)	57 (7.2%)	42 (34.1%)	
C_min_/dose [(mg l^−1^)/(mg d^−1^)]				**<0.001**
Median (IQR)	4.25 (2.25, 8.25)	3.88 (2.10, 6.93)	10.25 (5.4, 14.50)	
Range	0.08–51.0	0.08–51.0	0.40–42.50	
C_min_/dose level, *n* (%)^b^				**<0.000**
<1.25	108 (11.8%)	102 (12.8%)	6 (4.9%)	
[1.25, 12.5]	702 (76.5%)	626 (78.7%)	76 (61.8%)	
>12.5	108 (11.8%)	67 (8.4%)	41 (33.3%)	

*p* was calculated comparing oral administration with intravenous administration by the Mann–Whitney U test or chi-squared test, accordingly.

a The therapeutic index of VRC C_min_ is in accordance with the practice guideline for individualized medication of VRC reported by the Chinese Pharmacological Society. The lower limit of VRC C_min_ was set above 0.5 mg d^−1^ maintained-treatment response, and the higher limit was set as lowest concentration of hepatotoxicity.

b The therapeutic index of the VRC C_min_/dose ratio was calculated by VRC trough concentration divided by the most commonly used dose (400 mg d^−1^).

### Effects of Concomitant Glucocorticoids Administration on the C_min_/Dose Ratio of VRC

**FIGURE 1:**
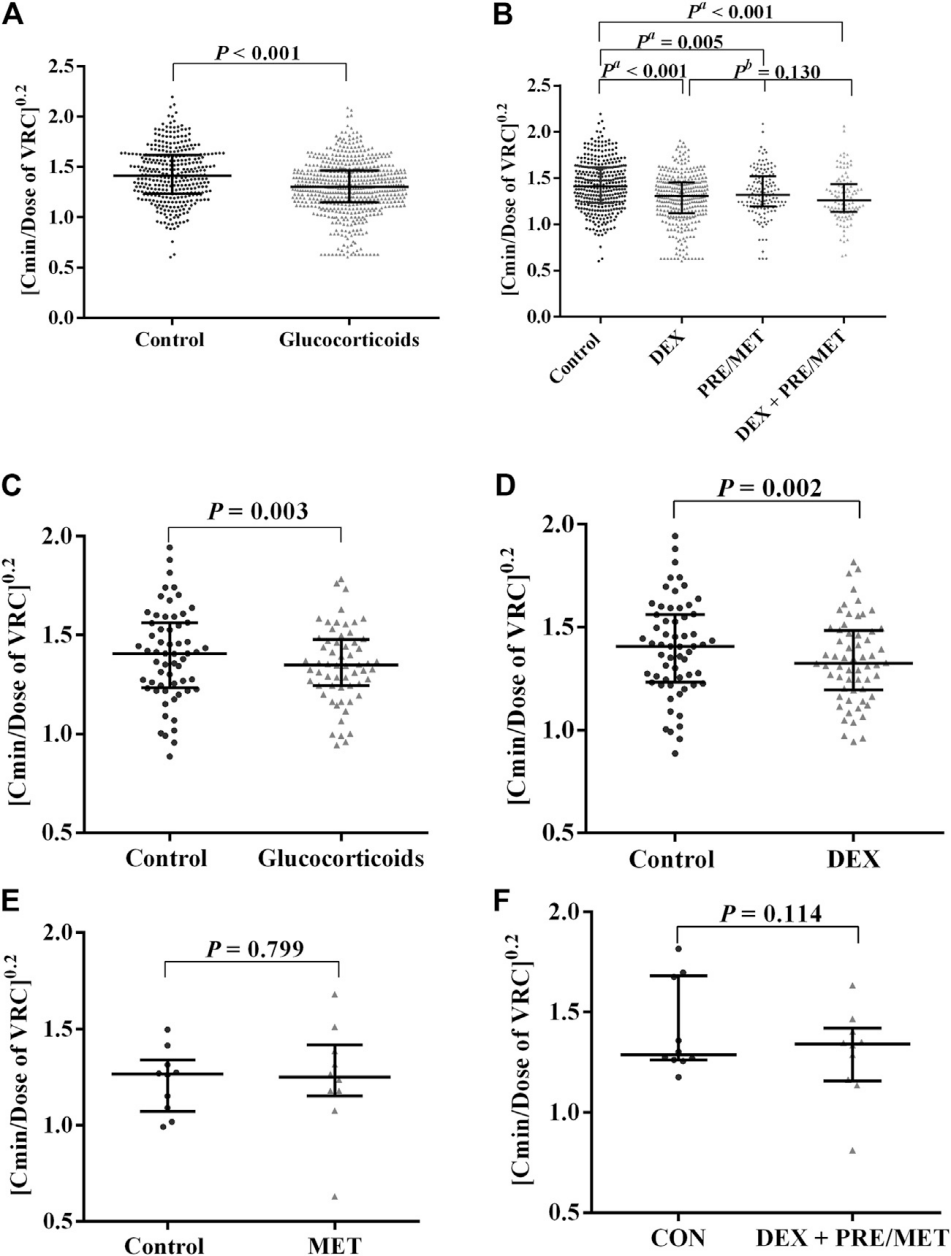
Effects of concomitant glucocorticoids administration on the C/D ratio of VRC. The data were non-normal distribution and expressed as median with interquartile range. The ordinate (C/D ratio of VRC) was processed to the power of 0.2. DEX was abbreviation of dexamethasone, PRE was abbreviation of prednisone or prednisolone, and MET was abbreviation of methylprednisolone. N represented the number of patients enrolled and n represented the number of VRC concentrations in the group. **(A)** showed that the C/D ratio of VRC was significantly higher in the control patients (*n* = 348) than the patients receiving glucocorticoids therapy simultaneously (*n* = 570) (*p* < 0.001). **(B)** showed the C/D ratio of VRC in the patients accompanying different kinds of glucocorticoids compared with the control patients (*n* = 348). Coadministration with DEX (*n* = 334, *p* < 0.001), PRE/MET (*n* = 134, *p* = 0.005), and DEX + PRE/MET (*n* = 102, *p* < 0.001) could all reduce the C/D ratio of VRC significantly, but there was no statistical difference among these three groups (*p* ^*b*^ = 0.130) (Supplemental Table S1). **(C)** showed that the C/D ratio of VRC was significantly higher in the control patients (*n* = 197) than the patients receiving glucocorticoids therapy simultaneously (*n* = 310) (N = 60, *p* = 0.003). **(D)** showed that the C/D ratio of VRC in the patients taking DEX (*n* = 236) was significantly lower than the control patients (*n* = 197) (N = 60, *p* = 0.002). **(E)** showed that the C/D ratio of VRC in the patients taking MET (*n* = 31) had no statistical difference compared with the control patients (*n* = 51) (N = 10, *p* = 0.799). **(F)** showed that the C/D ratio of VRC in the patients taking DEX + PRE/MET (*n* = 35) had no statistical difference compared with the control patients (*n* = 37) (N = 10, *p* = 0.114) (Supplemental Table S2).

As shown in Figure 1, 348 VRC C_min_ uncombined with glucocorticoids were designated as the control group and 570 VRC C_min_ concomitant with glucocorticoids were designated as the glucocorticoids group. Compared with the control group, concomitant with glucocorticoids decreased the VRC C_min_/dose ratio significantly (*p* < 0.01, Figure 1A). Dexamethasone (DEX group), prednisone/prednisolone or methylprednisolone (PRE/MET group), and dexamethasone and prednisone/prednisolone/methylprednisolone (DEX + PRE/MET group) all markedly decreased the VRC C_min_/dose ratio statistically (*p* ^*c*^ < 0.01, Figure 1B; Supplemental Table S1).

We further performed a paired test of the VRC C_min_/dose ratio with or without glucocorticoids to explore the effects of glucocorticoids on the VRC C_min_/dose ratio. 60 patients were monitored for VRC C_min_ underlying comedication with or without glucocorticoids. The number of VRC C_min_ measured in each patient ranged from 2 to 35, for a total of 507 concentrations included (see Supplemental Table S2). The paired test confirmed again that VRC C_min_/dose was reduced by glucocorticoids with statistical significance (*p* = 0.003, Figure 1C). As for the effects of different kinds of glucocorticoids on VRC, dexamethasone could decrease the median of the VRC C_min_/dose ratio by about 21.9% (*p* = 0.002, Figure 1D). These results verified that the combination of glucocorticoids could reduce the VRC C_min_/dose again.

### The Effects of Glucocorticoids on Influencing Probability of the Therapeutic Window of VRC

Based on our results, glucocorticoids can affect VRC C_min_/dose. We further explored the effects of glucocorticoids on the probability of the therapeutic window of VRC. It was found that the proportion of subtherapeutic VRC C_min_/dose were all increased after DEX or PRE/MET or DEX + PRE/MET administrations, although only the DEX group showed statistical significance (*p* < 0.001, Table 3). The groups with DEX and PRE/MET decreased the percentage of supratherapeutic VRC C_min_/dose (*p* < 0.001 and *p* = 0.005, Table 3). These results emphasized that combination with glucocorticoids would increase the proportion of VRC subtherapeutic concentration leading to poor treatment response. Therefore, more attention should be paid to clinical efficacy rather than the safety of VRC when combined with glucocorticoids in clinical therapy.

**TABLE 3:** The effect of glucocorticoids on influencing probability of the therapeutic window of VRC.

Group	C_min_/dose level *n* (%)			*p* ^*a*^	*p* ^*b*^	*p* ^*c*^	*p* ^*d*^
	Subtherapeutic [<1.25 (mg l^−1^)/(mg d^−1^)]	Therapeutic [1.25, 12.5] (mg l^−1^)/(mg·d^−1^)	Supratherapeutic [>12.5 (mg l^−1^)/(mg d^−1^)]				
Non-comedication with glucocorticoids (N = 348)	26 (7.5%)	256 (73.6%)	66 (19.0%)				
Concomitant with glucocorticoids (N = 570)							
DEX (N = 334)	55 (16.5%)	259 (77.5%)	20 (6.0%)	**˂0.001**	**˂0.001**	0.247	**˂0.001**
PRE/MET (N = 134)	14 (10.5%)	109 (81.3%)	11 (8.2%)	**0.012**	0.356	0.077	**0.005**
DEX + PRE/MET (N = 102)	13 (12.8%)	78 (76.5%)	11 (10.8%)	0.058	0.106	0.608	0.072

DEX: dexamethasone; PRE: prednisone/prednisolone; and MET: methylprednisolone.

*p* ^a^ was calculated comparing the group of concomitants with DEX or PRE/MET or DEX + PRE/MET with the group of non-comedication with glucocorticoids by the chi-squared test.

*p* ^*b–d*^ were the values of subtherapeutic/therapeutic/supratherapeutic C_min_/dose level compared to the group of concomitants with DEX or PRE/MET or DEX + PRE/MET and the group of non-comedication with glucocorticoids by the chi-squared test, respectively.

### Effects of CYP450 Polymorphisms on VRC

**FIGURE 2:**
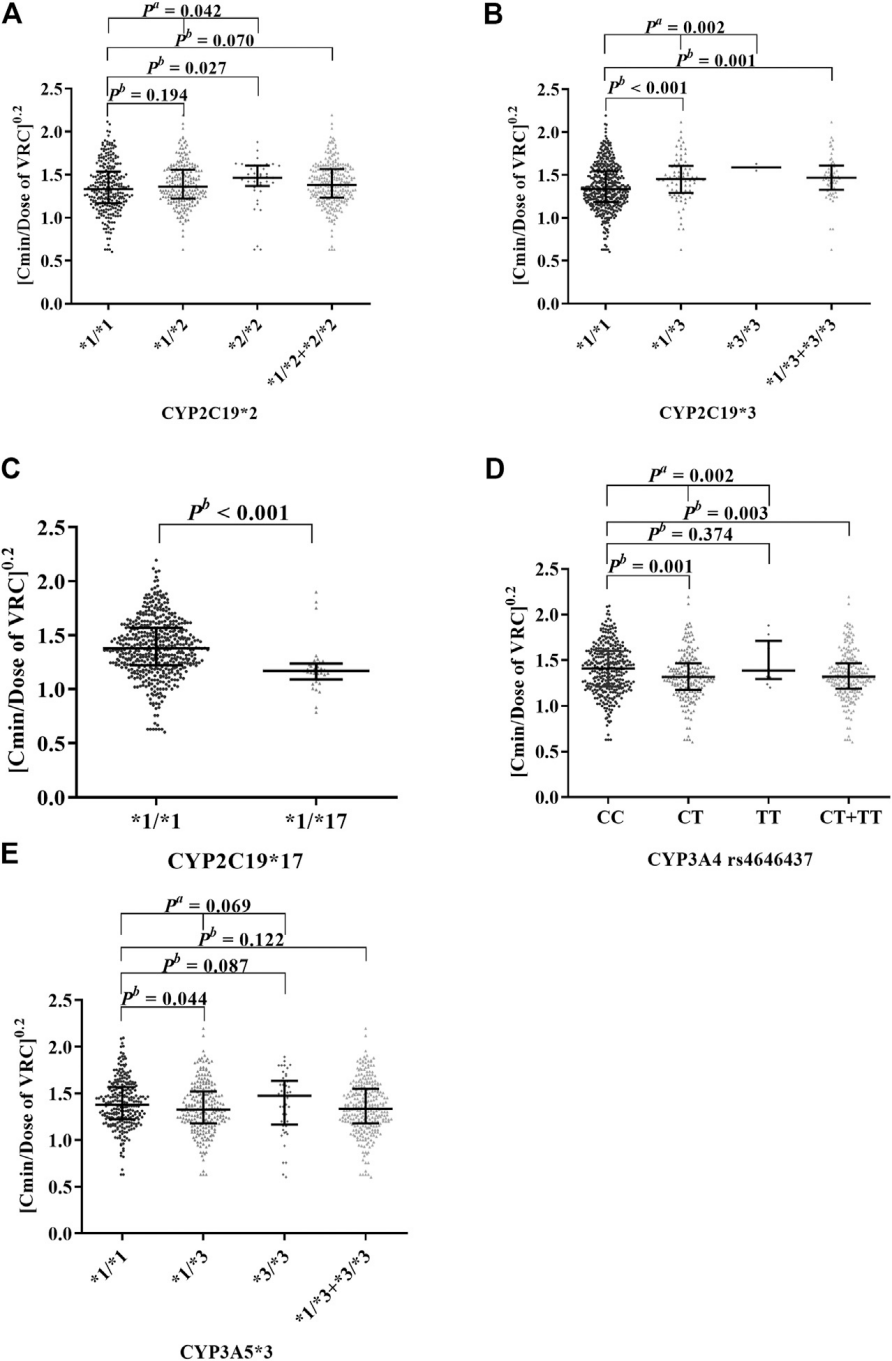
Effects of cytochrome P450 polymorphisms on the C/D ratio of VRC. The data were non-normal distribution and expressed as median with interquartile range. The ordinate (C/D ratio of VRC) was processed to the power of 0.2. Data showed in Supplemental Table S3. **(A)** showed that the comparison between CYP2C19*1/*1 (*n* = 286), CYP2C19*1/*2 (*n* = 228), and CYP2C19*2/*2 (*n* = 41) was significant (*p* ^*a*^ = 0.042). **(B)** showed that the CYP2C19*1/*3 + CYP2C19*3/*3 (*n* = 83) can increase the C/D ratio of VRC significantly compared to CYP2C19*1/*1 (*n* = 472, *p* ^*b*^ = 0.001). **(C)** showed that the CYP2C19*1/*17 (*n* = 35) can reduce the C/D ratio of VRC significantly compared to CYP2C19*1/*1 (*n* = 520, *P* ^*b*^ < 0.001). **(D)** showed that the comparison between *CYP3A4* genotype CC (*n* = 338) and CT + TT (*n* = 217) had statistical differences (*P* ^*b*^ = 0.003). (E) showed that the comparison between CYP3A5*1/*1 (*n* = 267), CYP3A5*1/*3 (*n* = 240), and CYP3A5*3/*3 (*n* = 48) was insignificant (*P* ^*a*^ = 0.069).

After clarifying the influences of glucocorticoids on VRC concentration, we explored whether the effects of glucocorticoids on VRC were related to CYP450s at first. We analyzed effects of *CYP2C19*, *CYP3A4*, and *CYP3A5* polymorphisms on the C_min_/dose ratio of VRC in 159 patients (N = 555) (shown in Figure 2; Supplemental Table S3). Allelic mutations of CYP2C19*2 (rs4244285) (*p* = 0.042, Figure 2A) and CYP2C19*3 (rs4986893) (*p* = 0.002, Figure 2B) both increased the C_min_/dose ratio of VRC, while allelic mutations of CYP2C19*17 (rs12248560) (*p* < 0.001, Figure 2C) and *CYP3A4* (rs4646437) (*p* = 0.002, Figure 2D) could both decrease the VRC C_min_/dose ratio statistically, despite the large inter-individual differences. It should be noted that mutants of CYP3A5*3 (rs776746, Figure 2E) showed no statistical effects (*p* = 0.069). In addition, no variant of CYP3A4*22 (rs 35599367) was detected in the present population. Our results suggested that allelic mutations of *CYP2C19*, *CYP3A4*, and *CYP3A5* can indeed affect VRC C_min_/dose, but different SNPs of CYP450 have different effects.

### Effects of CYP450 Polymorphisms on Glucocorticoids Reduced the C_min_/Dose Ratio and Probability of the Therapeutic Window of VRC

We further explored the interactions between glucocorticoids and CYP450 polymorphisms on the C_min_/dose ratio of VRC. Exception for CYP2C19*1/*3 and CYP2C19*3/*3, comedication with glucocorticoids reduced the C_min_/dose ratio of VRC significantly at each genotype compared with non-comedication groups (*p* < 0.05, Table 4). These results further confirmed that comedication with glucocorticoids could reduce the VRC C_min_/dose ratio. As shown in Table 4, mutants of CYP2C19*17 (*p* < 0.001) and CYP3A5*3 (*p* = 0.039) could reduce the C_min_/dose of VRC, while mutant of CYP2C19*3 (*p* = 0.003) could increase the C_min_/dose of VRC significantly in comedication with the glucocorticoids group. The above results indicated that the effects of CYP450 polymorphisms on VRC C_min_ were inconsistent and complex and the effects of DDIs between glucocorticoids and VRC played a major role in VRC C_min_ instead of CYP450 polymorphisms. Thus, more attention should be paid to the effects of DDIs between glucocorticoids and VRC rather than genetic polymorphisms when VRC was used.

**TABLE 4:** Effects of candidate SNPs on C_min_/dose of VRC in comedication or non-comedication with glucocorticoids.

Key haplotype	SNPs	Genotype	N	Comedication with glucocorticoids (N = 319)		*p* ^a^	Non-comedication with glucocorticoids (N = 236)		*p* ^a^	*p* ^b^	*p* ^c^
				N	C_min_/dose [(mg l^−1^)/(mg d^−1^)], median (IQR)		N	C_min_/dose [(mg l^−1^)/(mg d^−1^)], median (IQR)			
CYP2C19*2	rs4244285					0.052			0.572		0.798
CYP2C19*1/*1		GG	286	161	3.50 (1.91, 6.63)		125	5.67 (2.54, 13.63)		**˂0.001**	
CYP2C19*1/*2 + CYP2C19*2/*2		GA + AA	269	158	4.38 (2.48, 10.89)		111	6.50 (3.30,11.00)		**˂0.001**	
CYP2C19*3	rs4986893					**0.003**			0.106		0.578
CYP2C19*1/*1		GG	472	275	3.68 (2.08, 6.75)		197	5.67 (2.76, 11.98)		**0.003**	
CYP2C19*1/*3 + CYP2C19*3/*3		GA + AA	83	44	6.24 (3.55, 8.56)		39	7.20 (4.17,13.13)		0.106	
CYP2C19*17	rs12248560					**˂0.001**			0.713		**0.037**
CYP2C19*1/*1		CC	520	293	4.25 (2.50, 7.43)		227	6.40 (2.90, 11.75)		**˂0.001**	
CYP2C19*1/*17		CT	35	26	1.99 (1.29, 2.37)		9	3.38 (2.76, 17.88)		**0.001**	
CYP3A4	rs4646437					0.054			0.400		0.608
—		CC	338	170	4.55 (2.25, 8.52)		168	7.00 (2.96, 12.09)		**˂0.001**	
—		CT + TT	217	149	3.75 (2.15, 5.63)		68	5.28 (2.58, 12.13)		**0.002**	
CYP3A5*3	rs776746					**0.039**			0.610		0.159
CYP3A5*1/*1		GG	267	144	4.53 (2.40, 7.24)		123	5.74 (2.91, 11.00)		**0.015**	
CYP3A5*1/*3 + CYP3A5*3/*3		AG + AA	288	175	3.67 (1.86, 6.75)		113	6.90 (2.76, 13.25)		**˂0.001**	

a *p* was calculated comparing the mutant type with the wild type by the Mann–Whitney U test.

b *p* was calculated comparing between comedication with glucocorticoids and non-comedication with glucocorticoids in the same genotype by the Mann–Whitney U test.

c *p* was calculated comparing interaction between SNPs and glucocorticoids on the VRC C/D ratio by the Scheirer–Ray–Hare test.

Moreover, the two-factor nonparametric analysis of variances further suggested that only CYP2C19*17 genotypes had a significant interaction with glucocorticoids (*p* = 0.037, Table 4), which meant glucocorticoids had a more noteworthy effect on reducing the VRC C_min_/dose ratio in patients with CYP2C19*1/*17 genotype. Whereas, CYP2C19*2 mutation could increase the proportion of VRC C_min_ in the therapeutic window under comedication with glucocorticoids statistically (*p* = 0.030, Supplemental Table S4), and *CYP3A4* mutant decreased the proportion of VRC C_min_ in the supratherapeutic window (*p* = 0.033, Supplemental Table S4).

## Discussions

VRC is widely used in hematology, ICU, pneumology, and some other departments. The samples in our study were mainly collected from the hematology department. VRC is a first-line regimen in clinical preventions and treatments of invasive *Aspergillosis* infections recommended by the guidelines of the European Society of Clinical Microbiology and Infectious Diseases. In practical application, VRC is often inevitably coadministered with corticosteroids, proton pump inhibitors (PPIs), immunosuppressants, and other drugs, which lead to large individual differences. As a result, TDM-directed dose adjustment of VRC was recommended by guidelines (Moriyama et al., 2017). Although the proportion of the therapeutic VRC C_min_/dose ratio was higher in the present study than the previous literature (Cabral-Galeano et al., 2015; Zhou et al., 2020), there was still 22.2% (204 of 918) of VRC C_min_ in the subtherapeutic or supratherapeutic window. Therefore, it has great significance to clarify the influencing factors of VRC concentrations and conduct TDM detection for VRC. VRC can be administered orally or intravenously. Oral administration of VRC is more convenient and the bioavailability of VRC is over 90% because VRC can be absorbed quickly and thoroughly (Purkins et al., 2003; Theuretzbacher et al., 2006). Thus, VRC was mostly administered orally in clinical practice, which was consistent with the characteristics of our data and previous reports (Zeng et al., 2020).

The VRC C_min_ can be affected by numerous factors, among which CYP450 polymorphisms and DDIs can cause greater individual differences of VRC. It was reported that the pharmacokinetic values (AUC and C_max_) of VRC were changed to various degrees when combined with several PPIs (Qi et al., 2017). Coadministration of rifampicin was found to appreciably reduce VRC concentrations (Dolton et al., 2012). Since the combination of glucocorticoids and VRC is very common in clinical practice, the DDIs of VRC was focused on glucocorticoids in our research.

Although VRC could be metabolized by various CYP450 enzymes induced by corticosteroid (Li et al., 2017), the specific interference effect of glucocorticoids on the concentration and pharmacokinetics of VRC is still controversial (Dolton et al., 2012; Gautier-Veyret et al., 2015; Li et al., 2017; Imataki et al., 2018; Blanco-Dorado et al., 2020). Hence, the effects of glucocorticoid type and dosage on VRC remains to be further studied. Our results showed that combination with glucocorticoids could significantly reduce VRC C_min_/dose by both paired and unpaired tests. We further analyzed the effects of different kinds of glucocorticoids on VRC. Though the effect of dexamethasone in reducing VRC C_min_/dose was more obvious, there was no difference in the effect of different kinds of glucocorticoids, which was inconsistent with literature reports (Dolton et al., 2012; Li et al., 2017). In paired tests, we found that only the effect of dexamethasone on VRC C_min_/dose was significant, other types of glucocorticoids showed no statistical difference or cannot be analyzed because of the small sample size. In addition, glucocorticoids also decreased the proportion of VRC supratherapeutic C_min_/dose and increased the ratio of VRC subtherapeutic C_min_/dose, indicating that more attention should be paid to clinical efficacy rather than the safety of VRC when combined with glucocorticoids. On the whole, we verified glucocorticoids can reduce VRC C_min_/dose.

Since that glucocorticoids are inducers of CYP450 enzymes and VRC is mainly metabolized through CYP450 enzymes, we further investigated whether CYP450s are involved in the influence of glucocorticoids on VRC. VRC is metabolized by CYP2C19, CYP3A4, and CYP3A5 enzymes and polymorphisms of CYP450 affect VRC concentration by changing the CYP450s enzymatic activity (He et al., 2015; Lin et al., 2018). Our results further confirmed the argument that allelic mutations of CYP2C19*2 and *3 made a higher C_min_/dose ratio of VRC, while the allelic mutations of CYP2C19*17 and *CYP3A4* produced a lower C_min_/dose ratio of VRC (He et al., 2015; Moriyama et al., 2017). The effect of CYP3A5*3 on the VRC C_min_/dose ratio was slight and not significant which we speculated, the reason may be that CYP3A5 was not the main metabolic enzyme of VRC (Moriyama et al., 2017).

In order to explore the synergistic effects of glucocorticoids and CYP450 polymorphisms, we conducted two-factor nonparametric interaction analysis by the Scheirer–Ray–Hare test. The results showed that CYP2C19*17 was the only allele interacting with glucocorticoids. The effect of CYP2C19*17 on the reduction of concentration was strengthened by glucocorticoids, which suggested that there is a synergistic effect between CYP2C19*17 gene mutation and glucocorticoids. Mutations in CYP2C19*2, *3, and *CYP3A4* led to decreased enzyme activity, which was opposite to the effect of glucocorticoids on the activity of CYP2C19 and CYP3A4, so the overall effects on VRC C_min_/dose of these two factors were counterweighed. The CYP3A5*3 mutant caused a decrease in the VRC C_min_/dose ratio when combined with glucocorticoids, while the effect was opposite in non-comedication with the glucocorticoids group. We speculate the reason was that the mutation of CYP3A5*3 reduced the activity of hepatic microsomal enzyme CYP3A5 (Moriyama et al., 2017), thus reducing glucocorticoids metabolism and increasing glucocorticoids plasma concentration, leading to greater influence on VRC concentration when CYP3A5*3 mutated. The above results reminded us that other confounding factors (such as DDIs) rather than the genotype should be emphasized. Regardless of SNP mutations, coadministration with glucocorticoids could reduce the C_min_/dose ratio of VRC. However, when glucocorticoids were not used in combination with VRC, CYP450 mutations did not produce a statistically significant impact on the VRC C_min_/dose ratio. Therefore, it was suggested that the influence on the VRC C_min_/dose ratio of glucocorticoids was more illustrious than that of gene polymorphisms. Therefore, we recommend TDM rather than gene detection for routine clinical application (Moriyama et al., 2017).

Limitations still exist in this research. This is a retrospective study. The sample size of different glucocorticoids was dissimilar. In the paring analysis of glucocorticoids in VRC concentration, the available sample size of prednisone/prednisolone was too small to be analyzed. What is more, there are other factors that need to be considered in clinical practice, and the effect of glucocorticoids on VRC was influenced by other combination drugs or clinical factors. We did not analyze the combined effects of multiple drugs’ coadministration on VRC concentration which needs to be further explored. Moreover, our results showed that the effects of glucocorticoids on VRC cannot be fully explained by CYP450 polymorphisms, and other possible mechanisms such as inflammation need further investigation. In the rat septic shock mode, glucocorticoids can relieve inflammation and reduce C-reactive protein and procalcitonin (Li et al., 2019). Researchers have found that the inflammatory state could increase the plasma concentration of VRC through metabolic reduction in immunocompromised patients (Naito et al., 2015). Whether the effect of glucocorticoids on the concentration of VRC is related to the inflammatory state or is more closely related to the CYP450 genotypes deserves further study.

## Conclusion

In conclusion, our study confirmed that glucocorticoids reduced the C_min_/dose level of VRC despite the SNPs of CYP2C19 *2, *3, *17, *CYP3A4*, and *CYP3A5.* Glucocorticoids and CYP2C19*17 polymorphisms had a synergistic effect on reducing the VRC C_min_/dose ratio. The results indicated us that when combined with glucocorticoids, we should pay attention to the possibility of invalidation of VRC, especially when CYP2C19*17 mutation exists.

## Data Availability

The datasets presented in this study can be found in online repositories. The names of the repository/repositories and accession number(s) can be found in the article/Supplementary Material.

## Abbreviations

ADR: adverse drug reactions; C/D ratio: C_min_/dose ratio; C_min_: plasma trough concentrations; CYP450: cytochrome P450; DDIs: drug–drug interactions; IQR: interquartile difference; SD: standard deviation; TDM: therapeutic drug monitoring; VRC: voriconazole.

## References

[B1] Ashbee H. R. Barnes R. A. Johnson E. M. Richardson M. D. Gorton R. Hope W. W. (2014). Therapeutic Drug Monitoring (TDM) of Antifungal Agents: Guidelines from the British Society for Medical Mycology. J. Antimicrob. Chemother. 69 (5), 1162–1176. 10.1093/jac/dkt508 24379304 PMC3977608

[B2] Blanco-Dorado S. Maronas O. Latorre-Pellicer A. Rodriguez J. M. Lopez-Vizcaino A. Gomez M. A. (2020). Impact of CYP2C19 Genotype and Drug Interactions on Voriconazole Plasma Concentrations: A Spain Pharmacogenetic-Pharmacokinetic Prospective Multicenter Study. Pharmacotherapy 40 (1), 17–25. 10.1002/phar.2351 31782536

[B3] Cabral-Galeano E. Ruiz-Camps I. Len-Abad O. Pou-Clave L. Sorde-Masip R. Meije-Castillo Y. (2015). Clinical Usefulness of Therapeutic Drug Monitoring of Voriconazole in a university Hospital. Enferm Infecc Microbiol. Clin. 33 (5), 298–302. 10.1016/j.eimc.2014.09.005 25459191

[B4] Chawla P. K. Nanday S. R. Dherai A. J. Soman R. Lokhande R. V. Naik P. R. (2015). Correlation of CYP2C19 Genotype with Plasma Voriconazole Levels: a Preliminary Retrospective Study in Indians. Int. J. Clin. Pharm. 37 (5), 925–930. 10.1007/s11096-015-0143-y 26024717

[B5] Chen K. Zhang X. Ke X. Du G. Yang K. Zhai S. (2018a). Individualized Medication of Voriconazole: A Practice Guideline of the Division of Therapeutic Drug Monitoring, Chinese Pharmacological Society. Ther. Drug Monit. 40 (6), 663–674. 10.1097/FTD.0000000000000561 30192314 PMC6250289

[B6] Chen Y. Ferguson S. S. Negishi M. Goldstein J. A. (2003). Identification of Constitutive Androstane Receptor and Glucocorticoid Receptor Binding Sites in the CYP2C19 Promoter. Mol. Pharmacol. 64 (2), 316–324. 10.1124/mol.64.2.316 12869636

[B7] Cojutti P. Candoni A. Forghieri F. Isola M. Zannier M. E. Bigliardi S. (2016). Variability of Voriconazole Trough Levels in Haematological Patients: Influence of Comedications with Cytochrome P450(CYP) Inhibitors And/or with CYP Inhibitors Plus CYP Inducers. Basic Clin. Pharmacol. Toxicol. 118 (6), 474–479. 10.1111/bcpt.12530 26572687

[B8] Dolton M. J. Ray J. E. Chen S. C. Ng K. Pont L. G. Mclachlan A. J. (2012). Multicenter Study of Voriconazole Pharmacokinetics and Therapeutic Drug Monitoring. Antimicrob. Agents Chemother. 56 (9), 4793–4799. 10.1128/AAC.00626-12 22751544 PMC3421881

[B9] Dvorak Z. Pavek P. (2010). Regulation of Drug-Metabolizing Cytochrome P450 Enzymes by Glucocorticoids. Drug Metab. Rev. 42 (4), 621–635. 10.3109/03602532.2010.484462 20482443

[B10] Eiden C. Cociglio M. Hillaire-Buys D. Eymard-Duvernay S. Ceballos P. Fegueux N. (2010). Pharmacokinetic Variability of Voriconazole and N-Oxide Voriconazole Measured as Therapeutic Drug Monitoring. Xenobiotica 40 (10), 701–706. 10.3109/00498254.2010.503814 20642349

[B11] Gautier-Veyret E. Fonrose X. Stanke-Labesque F. (2016). A Genetic Score Combining CYP450 2C19 and 3A4 Genotypes to Predict Voriconazole Plasma Exposure?. Int. J. Antimicrob. Agents 48 (2), 221–222. 10.1016/j.ijantimicag.2016.05.002 27318623

[B12] Gautier-Veyret E. Fonrose X. Stanke-Labesque F. (2017). Pharmacogenetics of Voriconazole: CYP2C19 but Also CYP3A4 Need to Be Genotyped. Clin. Pharmacol. Ther. 102 (2), 189. 10.1002/cpt.662 28182291

[B13] Gautier-Veyret E. Fonrose X. Tonini J. Thiebaut-Bertrand A. Bartoli M. Quesada J. L. (2015). Variability of Voriconazole Plasma Concentrations after Allogeneic Hematopoietic Stem Cell Transplantation: Impact of Cytochrome P450 Polymorphisms and Comedications on Initial and Subsequent Trough Levels. Antimicrob. Agents Chemother. 59 (4), 2305–2314. 10.1128/AAC.04838-14 25645831 PMC4356835

[B14] Geist M. J. Egerer G. Burhenne J. Mikus G. (2006). Safety of Voriconazole in a Patient with CYP2C9*2/CYP2C9*2 Genotype. Antimicrob. Agents Chemother. 50 (9), 3227–3228. 10.1128/AAC.00551-06 16940139 PMC1563530

[B15] He H. R. Sun J. Y. Ren X. D. Wang T. T. Zhai Y. J. Chen S. Y. (2015). Effects of CYP3A4 Polymorphisms on the Plasma Concentration of Voriconazole. Eur. J. Clin. Microbiol. Infect. Dis. 34 (4), 811–819. 10.1007/s10096-014-2294-5 25515945

[B16] Iber H. Chen Q. Sewer M. Morgan E. T. (1997). Regulation of Hepatic Cytochrome P450 2C11 by Glucocorticoids. Arch. Biochem. Biophys. 345 (2), 305–310. 10.1006/abbi.1997.0292 9308903

[B17] Imataki O. Yamaguchi K. Uemura M. Fukuoka N. (2018). Voriconazole Concentration Is Inversely Correlated with Corticosteroid Usage in Immunocompromised Patients. Transpl. Infect. Dis. 20 (4), e12886. 10.1111/tid.12886 29570914

[B18] Jin H. Wang T. Falcione B. A. Olsen K. M. Chen K. Tang H. (2016). Trough Concentration of Voriconazole and its Relationship with Efficacy and Safety: a Systematic Review and Meta-Analysis. J. Antimicrob. Chemother. 71 (7), 1772–1785. 10.1093/jac/dkw045 26968880 PMC4896404

[B19] Karlsson M. O. Lutsar I. Milligan P. A. (2009). Population Pharmacokinetic Analysis of Voriconazole Plasma Concentration Data from Pediatric Studies. Antimicrob. Agents Chemother. 53 (3), 935–944. 10.1128/AAC.00751-08 19075073 PMC2650527

[B20] Li T. Y. Liu W. Chen K. Liang S. Y. Liu F. (2017). The Influence of Combination Use of CYP450 Inducers on the Pharmacokinetics of Voriconazole: a Systematic Review. J. Clin. Pharm. Ther. 42 (2), 135–146. 10.1111/jcpt.12493 28177134

[B21] Li X. Li M. Liu L. Tian X. Liang Y. (2019). Protective Effects of Glucocorticoid on Liver Injury in a Rat Sepsis Model. Exp. Ther. Med. 18 (4), 3153–3160. 10.3892/etm.2019.7899 31572556 PMC6755462

[B22] Lin X. B. Li Z. W. Yan M. Zhang B. K. Liang W. Wang F. (2018). Population Pharmacokinetics of Voriconazole and CYP2C19 Polymorphisms for Optimizing Dosing Regimens in Renal Transplant Recipients. Br. J. Clin. Pharmacol. 84 (7), 1587–1597. 10.1111/bcp.13595 29607533 PMC6005582

[B23] Matoulkova P. Pavek P. Maly J. Vlcek J. (2014). Cytochrome P450 Enzyme Regulation by Glucocorticoids and Consequences in Terms of Drug Interaction. Expert Opin. Drug Metab. Toxicol. 10 (3), 425–435. 10.1517/17425255.2014.878703 24451000

[B24] Matsunaga T. Maruyama M. Matsubara T. Nagata K. Yamazoe Y. Ohmori S. (2012). Mechanisms of CYP3A Induction by Glucocorticoids in Human Fetal Liver Cells. Drug Metab. Pharmacokinet. 27 (6), 653–657. 10.2133/dmpk.dmpk-12-nt-018 22673009

[B25] Moriyama B. Obeng A. O. Barbarino J. Penzak S. R. Henning S. A. Scott S. A. (2017). Clinical Pharmacogenetics Implementation Consortium (CPIC) Guidelines for CYP2C19 and Voriconazole Therapy. Clin. Pharmacol. Ther. 102 (1), 45–51. 10.1002/cpt.583 27981572 PMC5474211

[B26] Naithani R. Kumar R. (2005). Voriconazole. Indian Pediatr. 42 (12), 1207–1212. 16424557

[B27] Naito T. Yamada T. Mino Y. Kawakami J. (2015). Impact of Inflammation and Concomitant Glucocorticoid Administration on Plasma Concentration of Triazole Antifungals in Immunocompromised Patients. Clin. Chim. Acta 441, 127–132. 10.1016/j.cca.2014.12.024 25542532

[B28] Purkins L. Wood N. Greenhalgh K. Allen M. J. Oliver S. D. (2003). Voriconazole, a Novel Wide-Spectrum Triazole: Oral Pharmacokinetics and Safety. Br. J. Clin. Pharmacol. 56 (Suppl. 1), 10–16. 10.1046/j.1365-2125.2003.01993.x 14616408 PMC1884314

[B29] Qi F. Zhu L. Li N. Ge T. Xu G. Liao S. (2017). Influence of Different Proton Pump Inhibitors on the Pharmacokinetics of Voriconazole. Int. J. Antimicrob. Agents 49 (4), 403–409. 10.1016/j.ijantimicag.2016.11.025 28159656

[B30] Sanguinetti M. Posteraro B. Beigelman-Aubry C. Lamoth F. Dunet V. Slavin M. (2019). Diagnosis and Treatment of Invasive Fungal Infections: Looking Ahead. J. Antimicrob. Chemother. 74 (Suppl. 2), ii27–ii37. 10.1093/jac/dkz041 31222314

[B31] Shao B. Ma Y. Li Q. Wang Y. Zhu Z. Zhao H. (2017). Effects of Cytochrome P450 3A4 and Non-genetic Factors on Initial Voriconazole Serum Trough Concentrations in Hematological Patients with Different Cytochrome P450 2C19 Genotypes. Xenobiotica 47 (12), 1121–1129. 10.1080/00498254.2016.1271960 27937048

[B32] Theuretzbacher U. Ihle F. Derendorf H. (2006). Pharmacokinetic/pharmacodynamic Profile of Voriconazole. Clin. Pharmacokinet. 45 (7), 649–663. 10.2165/00003088-200645070-00002 16802848

[B33] Thompson G. R. Lewis J. N. (2010). Pharmacology and Clinical Use of Voriconazole. Expert Opin. Drug Metab. Toxicol. 6 (1), 83–94. 10.1517/17425250903463878 19947892

[B34] Ullmann A. J. Aguado J. M. Arikan-Akdagli S. Denning D. W. Groll A. H. Lagrou K. (2018). Diagnosis and Management of Aspergillus Diseases: Executive Summary of the 2017 ESCMID-ECMM-ERS Guideline. Clin. Microbiol. Infect. 24 (Suppl. 1), e1–e38. 10.1016/j.cmi.2018.01.002 29544767

[B35] Yan M. Wu Z. F. Tang D. Wang F. Xiao Y. W. Xu P. (2018). The Impact of Proton Pump Inhibitors on the Pharmacokinetics of Voriconazole *In Vitro* and *In Vivo* . Biomed. Pharmacother. 108, 60–64. 10.1016/j.biopha.2018.08.121 30216801

[B36] Zeng G. Wang L. Shi L. Li H. Zhu M. Luo J. (2020). Variability of Voriconazole Concentrations in Patients with Hematopoietic Stem Cell Transplantation and Hematological Malignancies: Influence of Loading Dose, Procalcitonin, and Pregnane X Receptor Polymorphisms. Eur. J. Clin. Pharmacol. 76 (4), 515–523. 10.1007/s00228-020-02831-1 31932875

[B37] Zhou P. Y. Lim T. P. Tang S. Liew Y. Chua S. Lim L. (2020). The Utility of Voriconazole Therapeutic Drug Monitoring in a Multi-Racial Cohort in Southeast Asia. J. Glob. Antimicrob. Resist. 21, 427–433. 10.1016/j.jgar.2019.12.004 31846723

[B38] Zhou S. F. Zhou Z. W. Yang L. P. Cai J. P. (2009). Substrates, Inducers, Inhibitors and Structure-Activity Relationships of Human Cytochrome P450 2C9 and Implications in Drug Development. Curr. Med. Chem. 16 (27), 3480–3675. 10.2174/092986709789057635 19515014

