# Supplementary material for: Interactive Effects of Glucocorticoids and Cytochrome P450 Polymorphisms on the Plasma Trough Concentrations of Voriconazole
Source: Front Pharmacol. 2021 May 25;12:666296. doi: 10.3389/fphar.2021.666296 (PMC8185288; doi:10.3389/fphar.2021.666296)
Supplement: Supplementary file 1 [file DataSheet1.docx]

**Supplemental Table S1 Effect of concomitant medication of glucocorticoids on C_min_/dose of VRC**

| **Group** | **Number** | **C_min_/dose [(mg·L^-1^)/(mg·d^-1^)], Median (IQR)** | ***P* value** |
| --- | --- | --- | --- |
| Control | 348 | 5.58 (2.86, 11.0) | **˂0.001** *^a^* |
| Concomitant with glucocorticoids | 570 | 3.75 (1.98, 6.75) | 0.130 *^b^* |
| DEX | 334 | 3.79 (1.77, 6.53) | **˂0.001** *^c^* |
| PRE/MET | 134 | 4.04 (2.44, 8.13) | **0.005** *^c^* |
| DEX + PRE/MET | 102 | 3.20 (1.87, 6.06) | **˂0.001** *^c^* |

DEX: Dexamethasone; PRE: Prednisone/Prednisolone; MET: Methylprednisolone

*^a^ P* values was calculated comparing control group with concomitant glucocorticoids group by Mann-Whitney U test.

*^b^ P* values was calculated comparing among DEX group, PRE/MET group and DEX+PRE/MET group by Kruskal-Wallis test.

*^c^ P* values was calculated comparing control group with concomitant DEX group, or PRE/MET group, or DEX + PRE/MET group by Mann-Whitney U test.

**Supplemental Table S2 Effect of glucocorticoid on C_min_/dose of VRC in the same patient**

| **Concomitant medication** | **Number of concentrations** | **C_min_/dose *^a^* [(mg·L^-1^)/(mg·d^-1^)],**  **Median (IQR)** | ***P* value *^b^*** |
| --- | --- | --- | --- |
| **Glucocorticoids**  **(N = 60)** |  |  | **0.003** |
| No (n = 197) | 2 (1, 4) | 5.25 (2.41, 9.46) |  |
| Yes (n = 310) | 4 (2, 7) | 4.00 (1.78, 7.43) |  |
| **DEX (N = 60)** |  |  | **0.002** |
| No (n = 197) | 2 (1, 4) | 5.25 (2.41, 9.46) |  |
| Yes (n = 236) | 3 (1, 6) | 4.10 (2.45, 7.22) |  |
| **MET (N = 10)** |  |  | 0.799 |
| No (n = 51) | 4.5 (2.75, 7.25) | 3.24 (1.43, 4.35) |  |
| Yes (n=31) | 2 (1.75, 4.25) | 2.00 (1.75, 4.25) |  |
| **DEX + PRE/MET**  **(N = 10)** |  |  | 0.114 |
| No (n = 37） | 2.5 (1, 6.5) | 3.55 (3.18, 13.4) |  |
| Yes (n = 35) | 2.5 (1, 4.5) | 4.33 (2.07, 5.78) |  |

DEX: Dexamethasone; PRE: Prednisone/prednisolone; MET: Methylprednisolone

N was the number of patients enrolled, n represented the number of VRC concentrations in the group.

*^a^* If there are multiple values that satisfy the conditions for the same patient, the average value of Cmin/dose🞪1000 was taken for calculation.

*^b^ P* values was calculated comparing non-concomitant with concomitant glucocorticoids in the same patient by Wilcoxon Rank Sum test.

**Supplemental Table S3 The effect of candidate SNPs on** **C_min_/dose of VRC (N = 555)**

| **Key Haplotype** | **SNPs** | **Genotype** | **Number** | **C_min_/dose**  **[(mg·L^-1^)/(mg·d^-1^)],**  **Median (IQR)** | ***P* value** |
| --- | --- | --- | --- | --- | --- |
| CYP2C19*2 | rs4244285 | GG | 286 | 4.21 (2.18, 8.63) | **0.042 *^a^*** |
| （G>A） |  | GA | 228 | 4.68 (2.75, 9.20) |  |
|  |  | AA | 41 | 6.75(4.83, 10.71) |  |
| CYP2C19*3 | rs4986893 | GG | 472 | 4.25 (2.32, 8.80) | **0.002 *^a^*** |
| （G>A） |  | GA | 81 | 6.50 (3.60, 10.75) |  |
|  |  | AA | 2 | 10.25 |  |
|  |  | GA+AA | 83 | 6.60 (3.70, 11.00) | **0.001 *^b^*** |
| CYP2C19*17 | rs12248560 | CC | 520 | 4.99 (2.73, 9.50) | **˂0.001 *^a^*** |
| （C>T） |  | CT | 35 | 2.20 (1.55, 2.90) |  |
| CYP3A4 | rs4646437 | CC | 338 | 5.55 (2.59, 10.50) | **0.002 *^a^*** |
| （C>T） |  | CT | 207 | 3.98 (2.25, 6.75) |  |
|  |  | TT | 10 | 5.13 (3.65, 14.81) |  |
|  |  | CT+TT | 217 | 4.00 (2.35, 6.78) | **0.003 *^b^*** |
| CYP3A5*3 | rs776746 | GG | 267 | 5.00 (2.75, 9.50) | 0.069 ***^a^*** |
| （G>A） |  | GA | 240 | 4.11 (2.28, 8.15) |  |
|  |  | AA | 48 | 7.01 (2.16, 11.69) |  |

*^a^ P* values was calculated by Kruskal-Wallis test to compare Cmin/Dose among wild type, heterozygous mutation and homozygous mutation of SNPs.

*^b^* *P* values was calculated comparing mutant type with wild type by Mann-Whitney U test.

**Supplemental Table S4 The effect of candidate SNPs on influencing probability of the therapeutic window of VRC under comedication with glucocorticoids (N = 319)**

| **Group** | | **C_min_/dose level, n (%)** | | | ***P ^a^***  **value** | ***P ^b^* value** | ***P ^c^* value** | ***P ^d^* value** |
| --- | --- | --- | --- | --- | --- | --- | --- | --- |
|  |  | **Subtherapeutic [<1.25 (mg·L^-1^)/(mg·d^-1^)]** | **Therapeutic [1.25, 12.5]**  **(mg·L^-1^)/(mg·d^-1^)** | **Supratherapeutic [˃12.5 (mg·L^-1^)/(mg·d^-1^)]** |  |  |  |  |
| CYP2C19 *2 (N = 319) | | | | | **0.030** |  |  |  |
| rs4244285 | GG (N = 161) | 25 (15.5%) | 121 (75.2%) | 15 (9.3%) |  | **0.035** | **0.010** | 0.289 |
|  | GA+AA (N = 158) | 12 (7.6%) | 137 (86.7%) | 9 (5.7%) |  |  |  |  |
| CYP2C19 *3 (N = 319) | | | | | 0.525 |  |  |  |
| rs4986893 | GG (N = 275) | 33 (12.0%) | 223 (81.1%) | 19 (6.9%) |  | 0.800 | 0.837 | 0.350 |
|  | GA+AA (N = 44) | 4 (9.1%) | 35 (79.5%) | 5 (11.4%) |  |  |  |  |
| CYP2C19 *17 (N = 319) | | | | | 0.167 |  |  |  |
| rs12248560 | CC (N = 293) | 32 (10.9%) | 237 (80.9%) | 24 (8.2%) |  | 0.204 | >0.9999 | 0.239 |
|  | CT (N = 26) | 5 (19.2%) | 21 (80.8%) | 0 |  |  |  |  |
| CYP3A4 (N = 319) | | | | | 0.081 |  |  |  |
| rs4646437 | CC (N = 170) | 20 (11.8%) | 132 (77.6%) | 18 (10.6%) |  | >0.999 | 0.153 | **0.033** |
|  | CT+TT (N = 149) | 17 (11.4%) | 126 (84.6%) | 6 (4.0%) |  |  |  |  |
| CYP3A5 *3 (N = 319) | | | | | 0.197 |  |  |  |
| rs776746 | GG (N = 144) | 12 (8.3%) | 119 (82.6%) | 13 (9.1%) |  | 0.115 | 0.479 | 0.398 |
|  | AG+AA (N = 175) | 25 (14.3%) | 139 (79.4%) | 11 (6.3%) |  |  |  |  |

*P^a^* values was calculated comparing mutant type with wild type by Chi-Square test.

*P ^b-d^* was the value of subtherapeutic/therapeutic/supratherapeutic Cmin/dose level compared to the group of mutant type and the group of wild type by Chi-Square test respectively.

**Supplemental Table S5 The sequence of *CYP450* polymorphisms**

| **Key Haplotype**  **( SNPs)** | **Sequence** |
| --- | --- |
| *CYP2C19*2*  (rs4244285) | **CCAGAGCTTGGCATATTGTATC**TATACCTTTATTAAATGCTTTTAATTTAATAAATTATTGTTTTCTCTTAGATATGCAATAATTTTCCCACTATCATTGATTATTTCCC**[G/A]**GGAACCCATAACAAATTACTTAAAAACCTTGCTTTTATGGAAAGTGATATTTTGGAGAAAGTAAAAGAACACCAAGAATCGATGGACATCAACAACCCTCGGGACTTTATTGATTGCTTCCTGATCAAAATGGAGAAGGTAAAATGTTAACAAAAGCTTAGTTAT**GTGACTGCTTGCGTATTTGTGA** |
| *CYP2C19*3*  (rs4986893) | CACCCTGTGATCCCACTTTCATCCTGGGCTGTGCTCCCTGCAATGTGATCTGCTCCATTATTTTCCAGAAACGTTTCGATTATAAAGATCAGCAATTTCTTAACTTGATGGAAAAATTGAATGAAAACATCAGGATTGTAAGCACCCCCTG**[G/A]**ATCCAGGTAAGGCCAAGTTTTTTGCTTCCTGAGAAACCACTTACAGTCTTTTTTTCTGGGAAATCCAAAATTCTATATTGACCAAGCCCTGAAGTACATTTTTGAATACTACAGTCTTGCCTAGACAGCCATGGGGTGAATATCTGGAAAAGATGGCAAAGTTCTTTATTTTATGCACAGGAAATGAATATCCCAATATAGA**TCAGGCTTCTAAGCCCATTAGC** |
| *CYP2C19*17*  (rs12248560) | **CACTGAGCGTTTCCCCTCTG**CAGTGATGGAGAAGGGAGAACTCTTATTTTTTCTCATGAGCATCTCTGGGGCTGTTTTCCTTAGATAAATAAGTGGTTCTATTTAATGTGAAGCCTGTTTTATGAACAGGATGAATGTGGTATATATTCAGAATAACTAATGTTTGGAAGTTGTTTTGTTTTGCTAAAACAAAGTTTTAGCAAACGATTTTTTTTTTCAAATTTGTGTCTTCTGTTCTCAAAG**[C/T]**ATCTCTGATGTAAGAGATAATGCGCCACGATGGGCATCAGAAGACCTCAGCTCAAATCCCAGTTCTGCCAGCTATGAGCTGTGTGGCACCAACAGGTGTCCTGTTCTCCCAGGGTCTCCCTTTTCCCATTTGAAATATAAAAAATA**ACAATTCCTGCCTTCACGTG** |
| *CYP3A4*  (rs4646437) | **CCATGATAGGTGACAGAGATATGC**TGTGTTATTTCTATCTTGACTACCTACTATTTCTTGAACAGCAAGATTAATTTTGAGCTTCAGATTATGATTTGGGTTATTCTAGGAGACTGTAGTCCAATAGATAAAGGCAAAGAGATTAGGGCATTGAATTTTGTTCCTTTTATCCTTCAAAAGATGCACAAGGGGCTGCTGATCTCACTGCTGTAG**[C/T]**GGTGCTCCTTATGCATAGACCTGCCCTTGCTCAGCCACTGGCCTGAAAGAGGGGCAAAAGTCATAGAAGGAATGGCTTCCAGTTGAGAACCTTGATGTCTTTTACTCTTCTGGTTGGTAGAGAAAACTAGAATTGCTCCAGGTAAATTTTGCACATTCACAATGAATTTCTTTTTCTGTTTTTGTTTTGTTTTTCCTACAGCAGTCTTTCCATTCCTCATCCCAATTCTTGAAGTATTAAATATCTGTGTGTTTCCAAGAGAAGTTACAAATTTTTTAAGAAAATCTGTAAAAA**GGATGAAAGAAAGTCGCCTCG** |
| *CYP3A5*  *(*rs776746) | **GACTTAGTAGACAGATGACACAGC**TCTAGATGTCCATGGGCCCCACACCAACTGCCCTTGCAGCATTTAGTCCTTGTGAGCACTTGATGATTTACCTGCCTTCAATTTTTCACTGACCTAATATTCTTTTTGATAATGAAGTATTTTAAACATATAAAACATTATGGAGAGTGGCATAGGAGATACCCACGTATGTACCACCCAGCTTAACGAATGCTCTACTGTCATTTCTAACCATAATCTCTTTAAAGAGCTCTTTTGTCTTTCA**[A/G]**TATCTCTTCCCTGTTTGGACCACATTACCCTTCATCATATGAAGCCTTGGGTGGCTCCTGTGTGAGACTCTTGCTGTGTGTCACACCCTAATGAACTAGAACCTAAGGTTGCTGTGTGTCGTACAACTAGGGGTATGGATTACATAACATAATGATCAAAGTCTGGCTTCCTGGGTGTGGCTCCAGCTGCAGAATCGGGCTAGTGAAGTTTAATCAGCTCCGTTGTCCCCACACAGAACGTATGAA**GGTCAACTCCCTGTGCTGG** |
